# Radiomics features for assessing tumor-infiltrating lymphocytes correlate with molecular traits of triple-negative breast cancer

**DOI:** 10.1186/s12967-022-03688-x

**Published:** 2022-10-15

**Authors:** Guan-Hua Su, Yi Xiao, Lin Jiang, Ren-Cheng Zheng, He Wang, Yan Chen, Ya-Jia Gu, Chao You, Zhi-Ming Shao

**Affiliations:** 1 Department of Breast Surgery, Key Laboratory of Breast Cancer in Shanghai, Fudan University Shanghai Cancer Center, No. 270 Dong’an Road, Shanghai, 200032 People’s Republic of China; 2 Department of Oncology, Shanghai Medical College, Fudan University, Shanghai, 200032 China; 3 Institute of Science and Technology for Brain-Inspired Intelligence, Fudan University, Shanghai, 201203 China; 4 Division of Cancer and Stem Cell, School of Medicine at University of Nottingham, Nottingham, UK; 5 Department of Radiology, Fudan University Shanghai Cancer Center, No. 270 Dong’an Road, Shanghai, 200032 People’s Republic of China

**Keywords:** Radiomics, Tumor-infiltrating lymphocytes (TILs), Triple-negative breast cancer (TNBC), Tumor microenvironment (TME), Magnetic resonance imaging (MRI)

## Abstract

**Background:**

Tumor-infiltrating lymphocytes (TILs) have become a promising biomarker for assessing tumor immune microenvironment and predicting immunotherapy response. However, the assessment of TILs relies on invasive pathological slides.

**Methods:**

We retrospectively extracted radiomics features from magnetic resonance imaging (MRI) to develop a radiomic cohort of triple-negative breast cancer (TNBC) (n = 139), among which 116 patients underwent transcriptomic sequencing. This radiomic cohort was randomly divided into the training cohort (n = 98) and validation cohort (n = 41) to develop radiomic signatures to predict the level of TILs through a non-invasive method. Pathologically evaluated TILs in the H&E sections were set as the gold standard. Elastic net and logistic regression were utilized to perform radiomics feature selection and model training, respectively. Transcriptomics was utilized to infer the detailed composition of the tumor microenvironment and to validate the radiomic signatures.

**Results:**

We selected three radiomics features to develop a TILs-predicting radiomics model, which performed well in the validation cohort (AUC 0.790, 95% confidence interval (CI) 0.638–0.943). Further investigation with transcriptomics verified that tumors with high TILs predicted by radiomics (Rad-TILs) presented activated immune-related pathways, such as antigen processing and presentation, and immune checkpoints pathways. In addition, a hot immune microenvironment, including upregulated T cell infiltration gene signatures, cytokines, costimulators and major histocompatibility complexes (MHCs), as well as more CD8^+^ T cells, follicular helper T cells and memory B cells, was found in high Rad-TILs tumors.

**Conclusions:**

Our study demonstrated the feasibility of radiomics model in predicting TILs status and provided a method to make the features interpretable, which will pave the way toward precision medicine for TNBC.

**Supplementary Information:**

The online version contains supplementary material available at 10.1186/s12967-022-03688-x.

## Introduction

Triple-negative breast cancer (TNBC) is defined as a breast cancer subtype that lacks expression of the estrogen receptor (ER), progenitor receptor (PR) and human epidermal growth factor receptor type 2 (HER2) [1]. Due to the aggressive biological nature of TNBC and the lack of therapeutic targets, TNBCs are characterized by frequent local recurrence and visceral metastasis [1, 2].

Tumor-infiltrating lymphocytes (TILs) have been used as a biomarker of prognosis and therapeutic response in several cancer types [3, 4]. In breast cancer, TILs are most commonly found in TNBC [5, 6]. In a series of clinical trials and prospective studies, recurrence-free survival (RFS), disease-free survival (DFS) and overall survival (OS) outcomes were positively correlated with the quantity of TILs in TNBC tumors [5–9]. Lymphocyte-predominant breast cancer (LPBC) is considered to be a type of breast cancer that responds better to chemotherapy than non-lymphocyte-predominant breast cancer (non-LPBC) [10–12]. In recent years, immunotherapy, particularly the use of immune checkpoint blockades (ICBs), has produced favorable clinical benefits in patients with both early and advanced TNBC [13–15]. In addition to current biomarkers [programmed cell death-ligand 1 (PD-L1), tumor mutation burden (TMB) and microsatellite instability/deficient mismatch repair (MSI/dMMR)] [16, 17], TILs are expected to become another biomarker for predicting patient response to ICBs. Currently, TILs are evaluated through features exhibited by hematoxylin and eosin (H&E)-stained pathological slides obtained via invasive biopsy [3].

Radiomics is a method for extracting high-throughput features from medical images [18, 19]. These quantitative features could be analyzed with data from other observations to reflect the presence of significant genomic events, patients' response to therapy and prognosis, ultimately contributing to cancer diagnosis and treatment [18–24]. The noninvasive and reproducible nature of radiomics provides us with a favorable approach to predict clinicopathological variables. However, radiomics is limited by its poor interpretability.

In this article, we developed a radiomics signature to infer TILs status noninvasively and investigate the molecular biological significance of the radiomics signature, hoping to overcome the poor interpretability and facilitate the clinical utilization of radiomics for TNBC treatment optimization.

## Methods

### Cohorts and datasets

**Fig. 1:**
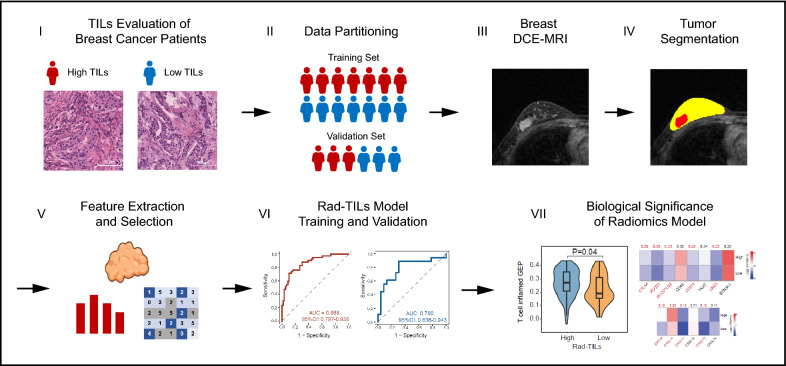
Schematic of the study. Tumor-infiltrating lymphocytes (TILs) densities were evaluated on H&E slides and were split into high and low TILs based on cut-off of 20%. Study cohort was randomly divided into training and validation cohort at a 7:3 ratio and similar high TILs proportion was kept in training and validation cohort. Regions of interest (ROIs) were segmented from the original breast MRI. Radiomics features were extracted from ROIs and were used to develop a TILs prediction model. Transcriptomics analysis was performed to further illustrate the radiomics model

**Fig. 2:**
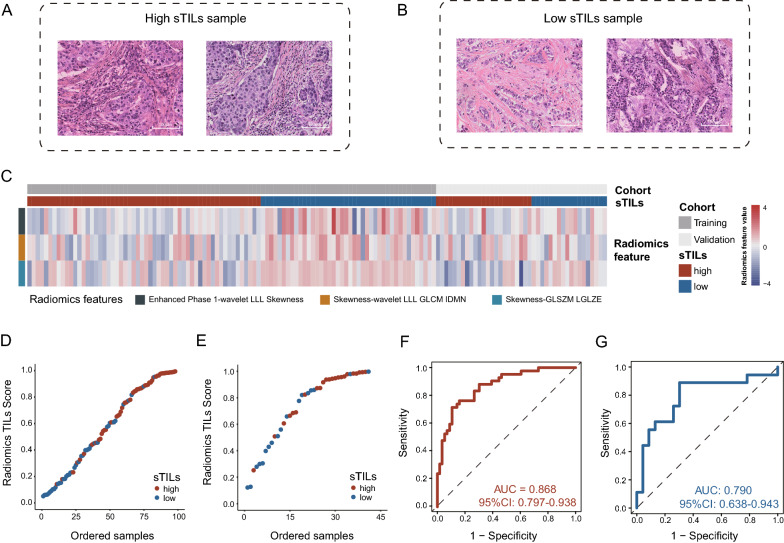
Training and validation of the TILs-predicting radiomics (Rad-TILs) model. **A**, **B** Representative TNBC pathological samples with high (**A**) and low (**B**) stromal tumor-infiltrating lymphocytes (sTILs). **C** Heatmap showing the distribution of selected radiomics feature value in high and low sTILs samples from the training and validation cohorts. **D**, **E** The correlation between sTILs status evaluated by pathologists (high and low sTILs) and TILs scores predicted by the radiomics model (Radiomics TILs score) in the training cohort (**D**) and validation cohort (**E**). **F**, **G** Receiver operating characteristic (ROC) curve of Rad-TILs model in the training cohort (**F**) and validation cohort (**G**)

We retrospectively enrolled 139 triple-negative breast cancer (TNBC) patients treated at the Fudan University Shanghai Cancer Center (FUSCC) from 1 August 2009 to 31 May 2015 with baseline dynamic contrast-enhanced magnetic resonance imaging (DCE-MRI) available who were suitable for radiomics analysis. In this TNBC radiomic cohort, transcriptomic data (n = 116) was also available. The framework of this study is presented as Fig. 1. The TNBC radiomics cohort (n = 139) was split into a training cohort (n = 98) and a validation cohort (n = 41) with a 7:3 ratio using a stratified randomization method to keep high and low TILs proportions similar in the two cohorts (Table 1). Quantification of stromal tumor-infiltrating lymphocytes (sTILs), fibrosis and necrosis were evaluated on pathological H&E staining area by two pathologists according to published guidelines [25, 26]. In this study, tumor-infiltrating lymphocytes (TILs) refer to sTILs unless otherwise specified. A percentage of sTILs ≥ 20% was defined as a high TILs level (Fig. 2A, B).

**Table 1:** Comparison of the basic information of the training and validation sets

Clinical characters	Level	Training set (n = 98)	Validation set (n = 41)	p value
Age (years, mean [SD])	–	54.28 (10.57)	54.61 (12.25)	0.872
Menopause (%)	False	31 (32.3)	13 (32.5)	1
	True	65 (67.7)	27 (67.5)	
T stage (%)	1	48 (49.0)	14 (34.1)	0.206
	2	46 (46.9)	26 (63.4)	
	3	4 (4.1)	1 (2.4)	
N stage (%)	0	56 (58.3)	27 (65.9)	0.226
	1	23 (24.0)	12 (29.3)	
	2	12 (12.5)	2 (4.9)	
	3	5 (5.2)	0 (0.0)	
Stromal TILs (%)	High	56 (57.1)	23 (56.1)	1
	Low	42 (42.9)	18 (43.9)	
Ki-67 [mean (SD)]	–	56.99 (21.90)	64.27 (26.14)	0.094
Molecular subtype (%)	BLIS	29 (29.6)	13 (31.7)	0.709
	IM	21 (21.4)	9 (22.0)	
	LAR	17 (17.3)	8 (19.5)	
	MES	16 (16.3)	3 (7.3)	
	Unknown	15 (15.3)	8 (19.5)	

*SD* standard deviation, *BLIS* basal-like immune-suppressed, *IM* immunomodulatory, *LAR* luminal androgen receptor, *MES* mesenchymal-like

### Magnetic resonance imaging (MRI) parameters

All the patients in this cohort underwent MRI with 1.5 T special breast magnetic resonance (Aurora Imaging Technology, Aurora Systems, Inc., Canada) and coils for breast. A series of cross-sectional images were obtained in prone position, including plain scan T2WI (TR 6680 ms, TE 68 ms, slice thickness 3 mm, slice spacing 1 mm), T1WI (TR 5 ms, TE 13 ms, slice thickness 3 mm, slice spacing 1 mm) and dynamic contrast-enhanced T1WI (TR 5 ms, TE 29 ms, slice thickness 1.1 mm, slice spacing 0 mm, FOV 360 × 360 mm). The contrast medium Gd-DTPA (0.2 mmol/kg, flow rate 2.0 ml/s) was injected 90 s after plain scan. Postcontrast images were obtained at 90, 180, 270, and 360 s after injection.

### Image preprocessing

In this study, the tumor regions of interest (ROIs) were delineated semiautomatically on the peak enhanced phase of CE-MRI by 3D Slicer software (https://www.slicer.org/). ROIs were placed on all slices that contained the whole tumor or the largest lesion (in the case of multicentric or multifocal tumors). To ensure reproducibility, some of the ROIs were initially delineated by two radiologists at FUSCC (C.Y. and D.D.Z. with 9 and 4 years of experience in breast MRI, respectively). The inter- and intra-observer reproducibility of the ROIs and radiomic feature extraction were initially analyzed with the CE-MRI data of 60 randomly selected patients in a blinded fashion by two radiologists. Additionally, one radiologist (C.Y. with 9 years of experience in breast MRI) repeated the ROI drawing twice with an interval of at least 1 month and generated radiomic features following the same procedure. Intraclass correlation coefficients (ICCs) were utilized to evaluate the intra- and interobserver agreement in terms of feature extraction. Inter- and intraobserver reproducibility and radiomic feature extraction achieved substantial agreement with ICC > 0.75 both among the ROIs from the two radiologists and between the ROIs from the same radiologist [27]. An ICC greater than 0.6 was considered a marker of satisfactory inter- and intra-observer reproducibility. On the premise of good consistency, whole ROI segmentation was completed by the more experienced radiologist in each layer of the MRI scan.

All other phases were co-registered into the first postcontrast phase of DCE-MRI through non-linear registration using the symmetric normalization algorithm [28], which was performed in the ANTs toolbox, to eliminate the spatial mismatches caused by motion artifact. Nonparametric nonuniformity normalization (N3) algorithm was applied for bias field correction [29]. Moreover, Z-Score Normalization algorithm was used for data normalization.

### Radiomics feature extraction

We performed a feature extraction process on DCE-MRI images (four phases) based on the open source Pyradiomics package V3.0, implemented in Python 3.6 [30], including shape features, first-order features, textural features, wavelet domain features and time domain features. For spatial domain features, 14 shape-based features were common to all phases, which describe the difference in shape between different types of tumors. Eighteen first-order features and 75 textural features were calculated from the four phases individually. First-order features describe the distribution of voxel intensities, and textural features were obtained based on 5 textural matrices to describe the radiological pattern of the ROI, including the gray level cooccurrence matrix (GLCM), gray level dependence matrix (GLDM), gray level run length matrix (GLRLM), gray level size zone matrix (GLSZM), and neighboring gray tone difference matrix (NGTDM). Moreover, wavelet domain features were extracted for each first order feature and textural feature by applying wavelet filtering to the original images, yielding 8 decompositions per level. In addition, for time domain features, the extracted sequential features were mainly composed of the mean, variance, kurtosis and skewness of the time-varying curve constructed based on feature values in four phases, for each first order, textural and wavelet domain feature. The specific number of features and the corresponding calculation formulae are described in detail in the Additional file 1.

### Model training and validation

Elastic net regression and logistic regression were utilized to select the most predictive radiomics features from the extracted features and to train machine learning model, respectively. Specifically, in the training cohort, we selected the most predictive radiomics features characterizing TILs levels with elastic net regression [31]. Then, the logistic regression was performed with the selected features to develop a TILs prediction signature referred to as Rad-TILs. The probability of high TILs predicted by radiomics model (p) was generated by the following formula:

$$ln\frac{p}{1-p}=\beta 0+\beta 1X1+\beta 2X2+\cdots +\beta pXp$$

In this study, $\beta 0$ = 0.9123828, $\beta 1$ = −0.6522518, $\beta 2$ = −0.9434133, $\beta 3$ = −1.5792121, *X1* = Enhanced-Phase-1-wavelet-LLL-Skewness, *X2* = Skewness-wavelet-LLL-GLCM-IDMN, *X3*= Skewness-GLSZM-LGLZE. Rad-TILs score was defined as the probability of high TILs predicted by radiomics model (p), where higher Rad-TILs score indicated a higher predictive probability of high TILs based on the three representative radiomics features. The efficiency of the prediction model was assessed by the receiver operating characteristic (ROC), specificity, sensitivity and accuracy in the validation cohort.

### Comparison of enriched pathways between groups

The Rad-TILs score was calculated by the Rad-TILs model in the subcohort of patients whose RNA-seq data were available (n = 116). Patients were separated into high- and low-Rad-TILs groups by the median Rad-TILs score in the model training process. We conducted differentially expressed gene (DEG) selection (“limma” package in R) [32] and KEGG pathway analysis (“clusterProfiler” package in R) [33]. Furthermore, we conducted gene set enrichment analysis (GSEA) using the KEGG and Reactome databases (“clusterProfiler” package in R) [33] to compare enriched pathways between high- and low-Rad-TILs patients.

### Comparison of immune infiltration in the microenvironment between groups

A previously published reference matrix of gene sets characterizing different immune cell populations suitable for breast cancer [34] was adopted in the present study. Single sample gene set enrichment analysis (ssGSEA) was used to calculate the immune cell abundance score in every patient (“GSVA” package in R) [35]. Then, the Wilcoxon test was utilized to compare the difference between the high- and low-Rad-TILs groups.

### Comparison of immune-related molecules between groups

Cytokines, costimulators, coinhibitors and major histocompatibility complexes (MHCs) were compared between the two groups by transcriptomic analysis. Furthermore, two gene signatures characterizing T cell inflammation status [36] and T cell cytolytic activity [37] were adopted to infer the T cell status in the two groups of patients.

### Statistical analysis

Student’s t test and Wilcoxon’s test were used to compare continuous variables. Prior to the comparisons, the normality of the distributions was tested with the Shapiro–Wilk test. Pearson’s chi-square test and Fisher’s exact test were employed for the comparison of unordered categorical variables. All the tests were two sided. P < 0.05 was regarded as indicating significance, and 0.05 < P < 0.1 was regarded as marginally significance unless otherwise stated. The false discovery rate (FDR) correction was used in multiple hypothesis testing to decrease false positive rates. All statistical analyses were performed with R software (version 4.0.3, http://www.R-project.org).

## Results

### TILs-related radiomics feature selection and prediction model establishment

We retrospectively curated 139 TNBC samples with preoperative DEC-MRI and post-operative H&E pathological slides to establish a TILs evaluation cohort. The intention of the study was split into two parts: generation of TILs prediction radiomics model and illustration of biological basis of the radiomics model (Fig. 1). A percentage of sTILs ≥ 20% was defined as high TILs level (Fig. 2A, B). With all the extracted radiomics features, we used elastic net regression to select the features that most closely correlated with tumor-infiltrating T lymphocytes (TILs) in the training cohort. The following three radiomics features were finally selected: Enhanced-Phase-1-wavelet-LLL-Skewness (spatial domain feature) that describes a first order imaging feature after applying wavelet filtering transformation of original first post-enhanced phase images, Skewness-wavelet-LLL-GLCM-IDMN (time domain feature) that depicts the variance pattern of a textural feature after wavelet filtering between each enhanced phase, and Skewness-GLSZM-LGLZE (time domain feature) that reflects the variance pattern of a textural feature between each enhanced phase (detailed description of radiomics features was presented in Additional file 1). The correlation between selected radiomics features and clinical characteristics was listed in Additional file 1: Table S1. In the training and validation cohorts, the three features presented a relatively lower value in tumors with high TILs (Fig. 2C). Then, the radiomic features were used as variables for logistic regression to build a prediction model. A predicted score reflecting the probability of high or low TILs (Radiomics TILs score, Rad-TILs score) for each patient was generated. We used the median of the Rad-TILs scores as a cutoff value to discriminate distinct Rad-TILs levels (Fig. 2D, E). The area under the receiver operating characteristic (ROC) curve (AUC) was 0.868 (95% CI 0.797–0.938) when predicting TILs in the training cohort, and the AUC was 0.790 (95% CI 0.638–0.943) in the validation cohort (Fig. 2F, G). In addition, the performance of the prediction model was tested for specificity (0.70), sensitivity (0.89) and accuracy (0.71) in the validation cohort.

### Immune-related pathways enriched in high Rad-TILs score patients

**Fig. 3:**
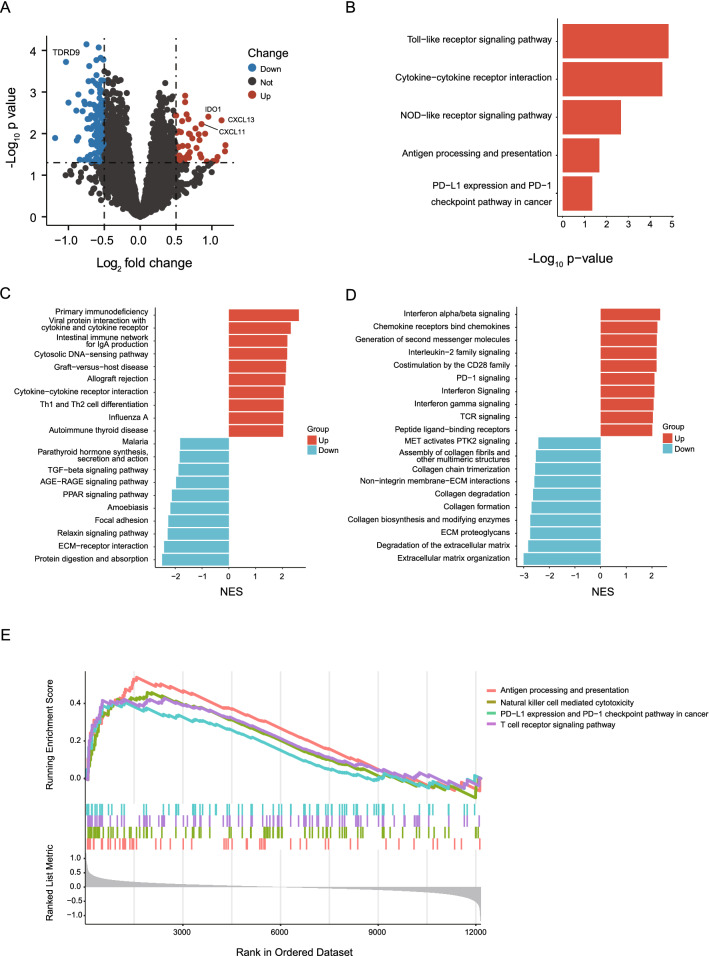
Immune related pathways enriched in the high TILs samples predicted by the radiomics model. **A** Differentially expressed genes between high and low TILs samples predicted by the radiomics model (high and low Rad-TILs). **B** Representative upregulated pathways in high Rad-TILs by KEGG enrichment analysis. **C** Top 10 upregulated and downregulated pathways in high Rad-TILs by GSEA based on KEGG database. **D** Top 10 upregulated and downregulated pathways in high Rad-TILs by GSEA based on Reactome databases. **E** Representative immune-related pathways enriched in high Rad-TILs by GSEA based on KEGG database

Cohort of patient with available radiomics and RNA-seq data was then used to investigate the transcriptomic difference between two sets of patients with distinct Rad-TILs levels. First, differentially expressed genes (DEGs) related to immunity, such as CXCL11, CXCL13, and IDO1, were discovered between the two groups (Fig. 3A). KEGG analysis inferred that several pathways correlated with the immune response, such as antigen processing (p = 0.02) and presentation and PD-L1 expression and PD-1 checkpoint pathway in cancer (p = 0.04), were significantly upregulated in high Rad-TILs patients (Fig. 3B). From the most upregulated and downregulated pathways summarized by GSEA based on the KEGG and Reactome databases, we found that the upregulated pathways were mainly enriched in immune response and immune modulation. However, the downregulated genes were difficult to categorize (Fig. 3C, D). Moreover, natural killer cell-mediated cytotoxicity and T cell receptor signaling pathway were upregulated in high Rad-TILs patients (Fig. 3E). Thus, we verified the different immune responses at the transcriptome level between the high- and low-Rad-TILs groups predicted by radiomics.

### A hot immune microenvironment in high Rad-TILs score patients

**Fig. 4:**
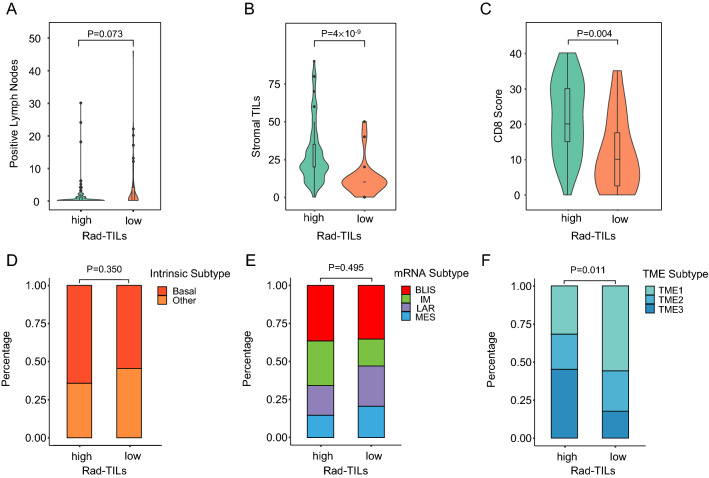
Clinicopathological and tumor microenvironmental (TME) characteristics of high and low Rad-TILs. **A**, **C** Lymph node status (**A**), sTILs quantification (**B**) and CD8 score (**C**) of high and low Rad-TILs. **D**–**F** Equivalent intrinsic subtypes (**D**), equivalent mRNA subtypes (**E**), and distinct TME subtypes (**F**) between high and low Rad-TILs. The p values were calculated using Wilcoxon test (for lymph node status, sTILs quantification and CD8 score) and Fisher’s exact test (for intrinsic subtypes, mRNA subtypes and TME subtypes)

We analyzed the correlation between Rad-TILs score levels and the clinicopathological characteristics of TNBC patients. High Rad-TILs score patients tended to have fewer pathologically positive lymph nodes (p = 0.073), but the difference was not statistically significant (Fig. 4A). Higher stromal TILs (sTILs) and immunohistochemistry (IHC) CD8 scores were detected in high Rad-TILs score patients (Fig. 4B, C). The intrinsic subtypes, mRNA subtypes, fibrosis and necrosis were equivalent between the two groups (Fig. 4D, E). Furthermore, tumor microenvironment (TME) cluster 3, which was proposed in our previous study to characterize the inflammatory immune status of TNBC [34], was significantly enriched in high Rad-TILs score patients (Fig. 4F).

**Fig. 5:**
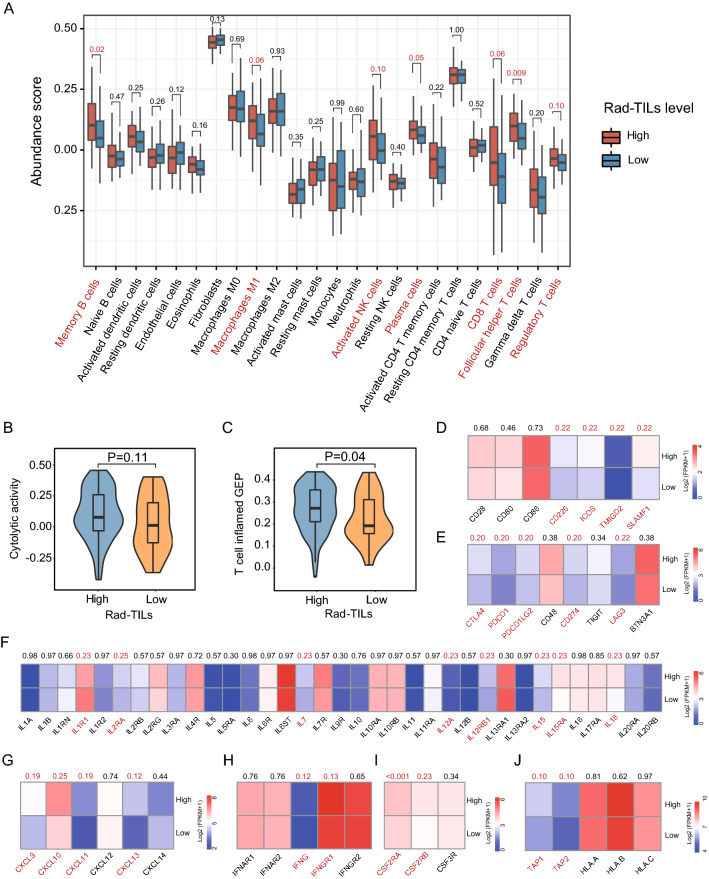
Inflamed TME in high Rad-TILs revealed by transcriptomics analysis. **A** Comparison of immune cell subpopulation between high and low Rad-TILs. The p values were calculated using Wilcoxon test. Specific p values were denoted on each cell type. **B**, **C** Expression of immune-related signatures in high and low Rad-TILs. Cytolytic activity (**B**) and T cell inflamed gene expression profiles (GEPs) (**C**) of two groups. The p values were calculated using Wilcoxon test. **D**, **J** Distinct expression of immune-related molecules on cell surface, including costimulators (**D**), coinhibitors (**E**) and major histocompatibility complex (**J**) between two groups. Distinct immune-related secretary molecules, including interleukins (**F**), chemokines (**G**), interferons (**H**) and colony-stimulating factors (**I**) between two groups. The p values calculated by Wilcoxon test were adjusted to false discovery rate (FDR) using the Benjamini–Hochberg procedure in multiple comparisons. Specific FDR values were denoted on each molecule

We also compared the difference in the composition of immune cells in the TME inferred by RNA-seq between high- and low-Rad-TILs score patients. Memory B cells, M1 macrophages, activated NK cells, plasma cells, CD8 T cells, follicular helper T cells and regulatory T cells were significantly or marginally significantly increased in the high Rad-TILs score group (Fig. 5A). Based on two published immune cell signatures, we found that patients with high Rad-TILs scores exhibited higher cytolytic activity and T cell inflamed gene expression profiles (Fig. 5B, C).

A relatively inflammatory TME in high Rad-TILs tumors was also indicated by the comparison of key molecules on the cell surface and cell-cell interactions. Several molecules expressed on the cell surface, including costimulators, coinhibitors and major histocompatibility complex (MHC), were highly expressed in high Rad-TILs score patient tumors (Fig. 5D, E, J). In addition, the levels of secreted immune-related cytokines, such as interleukins (ILs), colony-stimulating factors (CSFs), interferons (IFNs) and chemokines, were significantly elevated in the high group of patients (Fig. 5F–I), while the levels of transforming growth factors (TGFs) and tumor necrosis factors (TNFs) were equivalent between the two groups. Consequently, we established the relationship between opaque radiomics features and meaningful molecular features. Apart from distinct TILs levels, high Rad-TILs TNBC samples exhibited a hot immune microenvironment.

## Discussion

In the present study, we trained a TILs prediction model in the discovery cohort with a noninvasive radiomics method, which performed well in an additional validation cohort. In further investigation, we found a negative correlation between the Rad-TILs score and clinical risk factors, as well as the activated microenvironment exhibited in high Rad-TILs samples inferred by transcriptomics data, which supported and verified our initial finding. Significantly, radiomics combined with pathologic and transcriptomic data effectively reflected TILs status, and its potential mechanism was first reported in our study.

TILs play an important role in cancer biology and clinical oncology. TILs are regarded as biomarkers for immune infiltration and the prognosis of cancer patients and are promising potential biomarkers of patient response to immunotherapy. However, TILs quantification currently relies on manual evaluation of pathological slides, which is limited by the invasive method of specimen collection and time-consuming analysis approach.

Using a TNBC radiomics cohort with matched transcriptomic data, we established a three-feature radiomics signature, the Rad-TILs score, to noninvasively predict the level of sTILs, which are more commonly measured clinically than intratumoral TILs (iTILs). The prediction model performed well in the validation cohort with an AUC of 0.79. In addition, the high accuracy (0.71), sensitivity (0.89) and specificity (0.70) also validated our model. Prior to the present study, several studies explored the relationship between radiomics and TILs [38–46]. Consistent with our work, the range of AUC, sensitivity and specificity of these studies were 0.67–0.87, 0.63–0.89 and 0.56–0.91, respectively. Interestingly, we found that most of the studies achieved high sensitivity but low specificity, which was also testified in our results. We thus speculate that radiomics in TILs prediction is a method with high sensitivity and low specificity.

Although previous studies have reported the predictive value of radiomics features in TILs prediction, the biological characteristics of these crucial radiomics features or image subgroups were not fully investigated. A deeper investigation of distinct image subgroups provided novel insight into the interpretability of opaque radiomics features and correlated molecular features apart from TILs. In this study, we revealed the distinct TME features of patients with high and low lymphocyte infiltration predicted by radiomics (high and low Rad-TILs score) using matched RNA-seq, which demonstrated the unique value of multiomics in exploring the biological mechanism of radiomics features. First, we analyzed the DEGs between high- and low-Rad-TILs score patients and revealed that immune-related pathways were significantly enriched in the high-Rad-TILs score group. In addition, we compared the immune-related signatures, molecules and breast cancer subtypes between the two groups. Two representative signatures of the quantity and activity of T cells [36, 37, 47], several cytokines, immune checkpoint molecules and MHC molecules, were increased in the high Rad-TILs score group. The proportion of clusters characterizing immune inflammation [34] in the high Rad-TILs score group was also larger than that in the low Rad-TILs score group. Thus, it can be concluded that high Rad-TILs score tumors have an inflammatory immune microenvironment, and patients with high Rad-TILs scores are more likely to be sensitive to immunotherapy and have a better clinical outcome; however, the application of the Rad-TILs signature needs further validation in larger independent cohorts.

Moreover, we investigated the immune cells and TME subtypes in the two groups of patients. In addition to CD8-positive T cells, a variety of immune cells encompassing T helper cells, regulatory T cells, CD8 T cells, M1 macrophages, memory B cells and plasma cells aggregated in the high Rad-TILs score group. It has been reported that TILs comprise CD8^+^ cytolytic T cells, CD4^+^ helper T cells, CD20^+^ B cells and NK cells [3, 48, 49], which is consistent with our prediction. The important role of T cells has been well established [50, 51], and recent studies have shed light on the function of B cells and plasma cells in the homeostasis of the TME [52]. Specifically, B cells promote antitumor immunity through antibody and cytokine production, antigen presentation and their role in tertiary lymphoid structures formation [52]. Kroeger et al. discovered that plasma cells were strongly associated with CD8^+^ cytolytic T cells, and prognostic benefits were found only when coexisting with CD4^+^, CD20^+^ TILs and plasma cells in ovarian cancer [53]. Consistent with the results of previous studies, our study revealed the important role of B cells in the TME.

Several limitations still remain in our research. First, the prediction model was built and tested based on a single-center radiomics cohort. The universality of the model remains to be externally validated. In addition, the transcriptomic analysis inferred distinct TMEs between high- and low-Rad-TILs samples. However, the results need further phenotypic characterization and mechanistic investigation.

We propose two future directions for further studies. First, multicenter and prospective clinical trial are necessary to demonstrate the generalization of TILs prediction model. Second, recent study revealed distinct TILs infiltration phenotypes in cancer termed immune inflamed, immune desert and immune excluded, which indicated that lymphocytes infiltration pattern but not the density of TILs determined the activating status of anti-tumor immunity [54]. Whether these immune phenotypes were more valuable than TILs density as a predictive biomarker needs to be explored in future studies.

In conclusion, we established a TILs prediction model using radiomics features in a TNBC radiomics cohort and revealed the distinct composition and characteristics of the microenvironment in two groups of patients differentiated by our radiomics model. The radiomics model is promising for application in clinical practice and may become a noninvasive biomarker for therapeutic stratification and prognostic prediction among TNBC patients.

## Supplementary Information

**Additional file 1: Table S1.** Correlation between selected radiomics features and clinical characteristics listed in Table 1. **Table S2.** Feature categories used in this study. **Supplementary Methods.** Radiomics features calculation.

## Data Availability

The datasets generated and/or analyzed during the current study are available in the National Omics Data Encyclopedia (NODE), and can be viewed in NODE (http://www.biosino.org/node) by pasting the accession (OEP000155) into the text search box or through the URL:http://www.biosino.org/node/project/detail/OEP000155.

## Abbreviations

AUC: Area under the ROC curve
CSFs: Colony-stimulating factors
DEG: Differentially expressed gene
DFS: Disease-free survival
dMMR: Deficient mismatch repair
ER: Estrogen receptor
FDR: False discovery rate
FUSCC: Fudan University Shanghai Cancer Center
GSEA: Gene set enrichment analysis
H and E: Hematoxylin and eosin
HER2: Human epidermal growth factor receptor type 2
ICBs: Immune checkpoint blockades
IFNs: Interferons
IHC: Immunohistochemistry
ILs: Interleukins
iTILs: Intratumoral tumor-infiltrating lymphocytes
LPBC: Lymphocyte-predominant breast cancer
MHCs: Major histocompatibility complexes
MRI: Magnetic resonance imaging
MSI: Microsatellite instability
non-LPBC: Non-lymphocyte-predominant breast cancer
OS: Overall survival
PD-L1: Programmed cell death-ligand 1
PR: Progesterone receptor
RFS: Recurrence-free survival
RNA-seq: RNA sequencing
ROC: Receiver operating characteristic
ROIs: Regions of interest
ssGSEA: Single sample gene set enrichment analysis
sTILs: Stromal tumor-infiltrating lymphocytes
TGFs: Transforming growth factors
TILs: Tumor-infiltrating lymphocytes
TMB: Tumor mutation burden
TME: Tumor microenvironment
TNBC: Triple-negative breast cancer
TNFs: Tumor necrosis factors
