# Supplementary material for: Radiomics features for assessing tumor-infiltrating lymphocytes correlate with molecular traits of triple-negative breast cancer
Source: J Transl Med. 2022 Oct 15;20:471. doi: 10.1186/s12967-022-03688-x (PMC9571493; doi:10.1186/s12967-022-03688-x)
Supplement: Supplementary file 1 — Additional file 1: Table S1. Correlation between selected radiomics features and clinical characteristics listed in Table 1. Table S2. Feature categories used in this study. Supplementary Methods. Radiomics features calculation. [file 12967_2022_3688_MOESM1_ESM.docx]

**Table S1. Correlation between selected radiomics features and clinical characteristics listed in Table 1.**

|  | Feature 1 | | Feature 2 | | Feature 3 | |
| --- | --- | --- | --- | --- | --- | --- |
|  | R | p-value | R | p-value | R | p-value |
| Age | 0.15 | 0.0075 | -0.028 | 0.74 | 0.036 | 0.67 |
| Menopause (Ture/False) | - | 0.23 | - | 0.39 | - | 0.83 |
| T stage  (T1/T2/T3) | 0.14 | 0.10 | 0.03 | 0.69 | -0.003 | 0.93 |
| N stage  (N0/N1/N2/N3) | 0.05 | 0.52 | 0.09 | 0.27 | 0.07 | 0.36 |
| sTILs | -0.37 | <0.001 | -0.26 | 0.0024 | -0.26 | 0.0021 |
| Ki-67 | -0.1 | 0.22 | -0.062 | 0.47 | -0.084 | 0.33 |
| Subtype  (BLIS/IM/LAR/MES) | - | 0.32 | - | 0.28 | - | 0.47 |

Abbreviations: Feature 1, Enhanced-Phase-1-wavelet-LLL-Skewness; Feature 2, Skewness-wavelet-LLL-GLCM-IDMN; Feature 3, Skewness-GLSZM-LGLZE; BLIS, basal-like immune-suppressed; IM, immunomodulatory; LAR, luminal androgen receptor; MES, mesenchymal-like

**Table S2. Feature categories used in this study.**

| Feature classes | No. of features | Representative features |
| --- | --- | --- |
| Shape-based | 14 | Voxel Volume, Surface Area, Sphericity, …. |
| First Order Statistics | 18 (totally 72, 4 phases) | Energy, Entropy, Mean, …. |
| GLCM | 24 (totally 96, 4 phases) | Autocorrelation, Joint Average, Cluster Shade, …. |
| GLDM | 14 (totally 56, 4 phases) | Small Dependence Emphasis, Large Dependence Emphasis, Dependence Non-Uniformity |
| GLRLM | 16 (totally 64, 4 phases) | Short Run Emphasis, Long Run Emphasis, Run Length Non-Uniformity, …. |
| GLSZM | 16 (totally 64, 4 phases) | Small Area Emphasis, Large Area Emphasis, Size-Zone Non-Uniformity, …. |
| NGTDM | 5 (totally 20, 4 phases) | Coarseness, Contrast, Complexity, …. |
| Wavelet | 744 (totally 2976, 4 phases) | - |
| Time domain | 6696 | - |

GLCM = Gray Level Co-occurrence Matrix, GLDM = Gray Level Dependence Matrix, GLRLM = Gray Level Run Length Matrix, GLSZM = Gray Level Size Zone Matrix, NGTDM = Neighboring Gray Tone Difference Matrix.

The overall feature set was divided into spatial domain features and time domain features.

**Supplementary Methods. Radiomics features calculation**

**Spatial domain features**

Feature extraction was based on Pyradiomics V3.0 (voxel size: $0.7\times0.7\times1.5 {mm}^{3}$, 'binWidth': 25), implemented in Python 3.6. For spatial domain features, shape features were common to all phases (totally 14 features), with separate first order features and texture features for each phase (totally 372 features). Extracted texture features included Gray Level Co-occurrence Matrix [GLCM, directions: 13 angles in 3D (26-connectivity), distance: 1 voxel], Gray Level Dependence Matrix (GLDM, cutoff value $\alpha$: 0, distance: 1 voxel), Gray Level Run Length Matrix (GLRLM, directions: 13 angles in 3D), Gray Level Size Zone Matrix (GLSZM, directions: 13 angles in 3D), Neighboring Gray Tone Difference Matrix (NGTDM, neighborhood size: 3×3×3, distance: 1 voxel). For the GLCM, GLSZM and GLRLM, the value of a feature was calculated separately for each angle, after which the average of these values was obtained. Moreover, wavelet domain features were extracted for each first order features and texture features after applying wavelet filtering to the original images, yielding 8 decompositions per level (LLL, LLH, LHL, HLL, LHH, HLH, HHL, HHH) (totally 2976 features). The feature categories are listed in Table S1 and are described in detail as follows.

Shape Features (3D)

Shape-based features included descriptors of the three-dimensional size and shape of the ROI. These features are independent from the gray level intensity distribution in the ROI and are therefore only calculated on the non-derived image and mask.

Let:

- $N_{v}$ represent the number of voxels included in the ROI.
- $N_{f}$ represent the number of faces (triangles) defining the Mesh.
- $V$ the volume of the mesh in ${mm}^{3}$.
- $A$ the surface area of the mesh in ${mm}^{2}$.

1. Mesh Volume

$$V_{i}=\frac{Oa_{i}\cdot\left( Ob_{i}\times Oc_{i} \right)}{6}$$

$$V=\sum_{i=1}^{N_{f}} V_{i}$$

2. Voxel Volume

$$V_{voxel}=\sum_{k=1}^{N_{v}} V_{k}$$

3. Surface Area

$$A_{i}=\frac{1}{2}|a_{i}b_{i}\times a_{i}c_{i}|$$

$$A=\sum_{i=1}^{N_{f}} A_{i}$$

4. Surface Area to Volume ratio

$$surface to volume ratio=\frac{A}{V}$$

5. Sphericity

$$sphericity=\frac{\sqrt[3]{36\pi V^{2}}}{A}$$

6. Maximum 3D diameter

Maximum 3D diameter is defined as the largest pairwise Euclidean distance between tumor surface mesh vertices.

1. Maximum 2D diameter (Slice)

Maximum 2D diameter (Slice) is defined as the largest pairwise Euclidean distance between tumor surface mesh vertices in the row-column (generally the axial) plane.

1. Maximum 2D diameter (Column)

Maximum 2D diameter (Column) is defined as the largest pairwise Euclidean distance between tumor surface mesh vertices in the row-slice (usually the coronal) plane.

1. Maximum 2D diameter (Row)

Maximum 2D diameter (Row) is defined as the largest pairwise Euclidean distance between tumor surface mesh vertices in the column-slice (usually the sagittal) plane.

1. Major Axis Length

$$major axis=4\sqrt{\lambda_{major}}$$

2. Minor Axis Length

$$minor axis=4\sqrt{\lambda_{minor}}$$

3. Least Axis Length

$$least axis=4\sqrt{\lambda_{least}}$$

4. Elongation

$$elongation=\sqrt{\frac{\lambda_{minor}}{\lambda_{major}}}$$

5. Flatness

$$flatness=\sqrt{\frac{\lambda_{least}}{\lambda_{major}}}$$

First Order Features

First-order statistics describe the distribution of voxel intensities within the image region defined by the mask through commonly used and basic metrics.

Let:

- $X$ be a set of $N_{p}$ voxels included in the ROI.
- $\boldsymbol{P}(i)$ be the first order histogram with $N_{g}$ discrete intensity levels, where $N_{g}$ is the number of non-zero bins, equally spaced from 0 with a width defined in the binWidth parameter.
- $p(i)$ be the normalized first order histogram and equal to $\frac{\boldsymbol{P}(i)}{N_{p}}$.

1. Energy

$$energy=\sum_{i=1}^{N_{p}} {(\boldsymbol{X}\left( i \right)+c)}^{2}$$

2. Total Energy

$$total energy=V_{voxel}\sum_{i=1}^{N_{p}} {(\boldsymbol{X}\left( i \right)+c)}^{2}$$

3. Entropy

$$entropy=-\sum_{i=1}^{N_{g}} p(i){log}_{2}\left( p(i)+\epsilon\right)$$

4. Minimum

$$minimum=min(\boldsymbol{X})$$

5. 10th percentile

The ${10}^{th}$ percentile of $\boldsymbol{X}$.

1. 90th percentile

The ${90}^{th}$ percentile of $\boldsymbol{X}$.

1. Maximum

$$maximum=min(\boldsymbol{X})$$

2. Mean

$$mean=\frac{1}{N_{p}}\sum_{i=1}^{N_{p}} \boldsymbol{X}\left( i \right)$$

3. Median

The median gray level intensity within the ROI.

1. Interquartile Range

$$interquartile range=P_{75}-P_{25}$$

Here $P_{25}$ and $P_{75}$ are the ${25}^{th}$ and ${75}^{th}$ percentile of the image array, respectively.

1. Range

$$range=\max\left( \boldsymbol{X} \right)-min(\boldsymbol{X})$$

2. Mean Absolute Deviation (MAD)

$$MAD=\frac{1}{N_{p}}\sum_{i=1}^{N_{p}} \boldsymbol{|X}\left( i \right)-\bar{\boldsymbol{X}}|$$

3. Robust Mean Absolute Deviation (rMAD)

$$rMAD=\frac{1}{N_{10-90}}\sum_{i=1}^{N_{10-90}} \boldsymbol{|}\boldsymbol{X}_{\boldsymbol{10-90}}\left( i \right)-{\bar{\boldsymbol{X}}}_{10-90}|$$

4. Root Mean Squared (RMS)

$$RMS=\sqrt{\frac{1}{N_{p}}\sum_{i=1}^{N_{p}} {(\boldsymbol{X}\left( i \right)+c)}^{2}}$$

5. Skewness

$$skewness=\frac{\mu3}{\sigma^{3}}=\frac{\frac{1}{N_{p}}\sum_{i=1}^{N_{p}} {(\boldsymbol{X}\left( i \right)-\bar{\boldsymbol{X}})}^{3}}{{(\sqrt{\frac{1}{N_{p}}\sum_{i=1}^{N_{p}} {(\boldsymbol{X}\left( i \right)-\bar{\boldsymbol{X}})}^{2}})}^{3}}$$

6. Kurtosis

$$kurtosis=\frac{\mu4}{\sigma^{4}}=\frac{\frac{1}{N_{p}}\sum_{i=1}^{N_{p}} {(\boldsymbol{X}\left( i \right)-\bar{\boldsymbol{X}})}^{4}}{{(\sqrt{\frac{1}{N_{p}}\sum_{i=1}^{N_{p}} {(\boldsymbol{X}\left( i \right)-\bar{\boldsymbol{X}})}^{2}})}^{2}}$$

7. Variance

$$variance=\frac{1}{N_{p}}\sum_{i=1}^{N_{p}} {(\boldsymbol{X}\left( i \right)-\bar{\boldsymbol{X}})}^{2}$$

8. Uniformity

$$uniformity=\sum_{i=1}^{N_{g}} {p(i)}^{2}$$

Gray Level Co-occurrence Matrix (GLCM) Features

A Gray Level Co-occurrence Matrix (GLCM) of size $N_{g}\times N_{g}$describes the second-order joint probability function of an image region constrained by the mask and is defined as $\boldsymbol{P}(i,j|\delta,\theta)$. The ${(i,j)}^{th}$ element of this matrix represents the number of times the combination of levels $i$ and $j$ occur in two pixels in the image, that are separated by a distance of $\delta$ pixels along angle $\theta$. The distance $\delta$ from the center voxel is defined as the distance according to the infinity norm. For $\delta=1$, this results in 2 neighbors for each of 13 angles in 3D (26-connectivity) and for $\delta=2$ a 98-connectivity (49 unique angles).

Let:

- $\epsilon$ be an arbitrarily small positive number ($\approx{2.2\times10}^{-16}$).
- $\boldsymbol{P}(i,j)$ be the co-occurrence matrix for an arbitrary $\delta$ and $\theta$.
- $p(i,j)$ be the normalized co-occurence matrix and equal to $\frac{\boldsymbol{P}(i,j)}{\sum\boldsymbol{P}(i,j)}$.
- $N_{g}$ be the number of discrete intensity levels in the image.
- $p_{x}\left( i \right)=\sum_{j=1}^{N_{g}} \boldsymbol{P}(i,j)$ be the marginal row probabilities.
- $p_{y}\left( j \right)=\sum_{i=1}^{N_{g}} \boldsymbol{P}(i,j)$ be the marginal column probabilities.
- $\mu_{x}$ be the mean gray level intensity of $p_{x}$ and defined as $\mu_{x}=\sum_{i=1}^{N_{g}} p_{x}\left( i \right)i$.
- $\mu_{y}$ be the mean gray level intensity of $p_{y}$ and defined as $\mu_{y}=\sum_{j=1}^{N_{g}} p_{y}\left( j \right)j$.
- $\sigma_{x}$ be the standard deviation of $p_{x}$.
- $\sigma_{y}$ be the standard deviation of $p_{y}$.
- $p_{x+y}\left( k \right)=\sum_{i=1}^{N_{g}} \sum_{j=1}^{N_{g}} p(i,j)$, where $i+j=k$, and $k=2,3,\ldots,2N_{g}$.
- $p_{x-y}\left( k \right)=\sum_{i=1}^{N_{g}} \sum_{j=1}^{N_{g}} p(i,j)$, where $|i-j|=k$, and $k=0,1,\ldots,N_{g}-1$.
- $HX=-\sum_{i=1}^{N_{g}} p_{x}\left( i \right){log}_{2}(p_{x}\left( i \right)+\epsilon)$ be the entropy of $p_{x}$.
- $HY=-\sum_{j=1}^{N_{g}} p_{y}\left( j \right){log}_{2}(p_{y}\left( j \right)+\epsilon)$ be the entropy of $p_{y}$.
- $HXY=-\sum_{i=1}^{N_{g}} \sum_{j=1}^{N_{g}} p(i,j){log}_{2}(p(i,j)+\epsilon)$ be the entropy of $p(i,j)$.
- $HXY1=-\sum_{i=1}^{N_{g}} \sum_{j=1}^{N_{g}} p(i,j){log}_{2}(p_{x}\left( i \right)p_{y}\left( j \right)+\epsilon)$.
- $HXY2=-\sum_{i=1}^{N_{g}} \sum_{j=1}^{N_{g}} p_{x}\left( i \right)p_{y}\left( j \right){log}_{2}(p_{x}\left( i \right)p_{y}\left( j \right)+\epsilon)$.

1. Autocorrelation

$$autocorrelation=\sum_{i=1}^{N_{g}} \sum_{j=1}^{N_{g}} p(i,j)ij$$

2. Joint Average

$$joint average=\mu_{x}=\sum_{i=1}^{N_{g}} \sum_{j=1}^{N_{g}} p(i,j)i$$

3. Cluster Prominence

$$cluster Prominence=\sum_{i=1}^{N_{g}} \sum_{j=1}^{N_{g}} {(i+j-\mu_{x}-\mu_{y})}^{4}p(i,j)$$

4. Cluster shade

$$cluster shade=\sum_{i=1}^{N_{g}} \sum_{j=1}^{N_{g}} {(i+j-\mu_{x}-\mu_{y})}^{3}p(i,j)$$

5. Cluster Tendency

$$cluster tendency=\sum_{i=1}^{N_{g}} \sum_{j=1}^{N_{g}} {(i+j-\mu_{x}-\mu_{y})}^{2}p(i,j)$$

6. Contrast

$$\mathrm{contrast}=\sum_{i=1}^{N_{g}} \sum_{j=1}^{N_{g}} {(i-j)}^{2}p(i,j)$$

7. Correlation

$$correlation=\frac{\sum_{i=1}^{N_{g}} \sum_{j=1}^{N_{g}} p\left( i,j \right)ij-\mu_{x}\mu_{y}}{\sigma_{x}(i)\sigma_{y}(j)}$$

8. Difference Average

$$difference average=\sum_{k=0}^{N_{g}-1} kp_{x-y}(k)$$

9. Difference Entropy

$$difference entropy=\sum_{k=0}^{N_{g}-1} p_{x-y}\left( k \right){log}_{2}(p_{x-y}(k)+\epsilon)$$

10. Difference Variance

$$difference variance=\sum_{k=0}^{N_{g}-1} {(k-DA)}^{2}p_{x-y}\left( k \right)$$

11. Joint Energy

$$joint energy=\sum_{i=1}^{N_{g}} \sum_{j=1}^{N_{g}} {(p\left( i,j \right))}^{2}$$

12. Joint Entropy

$$joint entropy=-\sum_{i=1}^{N_{g}} \sum_{j=1}^{N_{g}} p(i,j){log}_{2}(p(i,j)+\epsilon)$$

13. Informational Measure of Correlation (IMC) 1

$$IMC1=\frac{HXY-HXY1}{max\{HX,HY\}}$$

14. Informational Measure of Correlation (IMC) 2

$$IMC2=\sqrt{1-e^{-2(HXY2-HXY)}}$$

15. Inverse Difference Moment (IDM)

$$IDM=\sum_{k=0}^{N_{g}-1} \frac{p_{x-y}\left( k \right)}{1+k^{2}}$$

16. Maximal Correlation Coefficient (MCC)

$$MCC=\sqrt{second largest eigenvalue of Q}$$

$$Q\left( i,j \right)=\sum_{k=0}^{N_{g}} \frac{p(i,k)p(j,k)}{p_{x}\left( i \right)p_{y}\left( k \right)}$$

17. Inverse Difference Moment Normalized (IDMN)

$$IDMN=\sum_{k=0}^{N_{g}-1} \frac{p_{x-y}\left( k \right)}{1+(\frac{k^{2}}{N_{g}^{2}})}$$

18. Inverse Difference (ID)

$$ID=\sum_{k=0}^{N_{g}-1} \frac{p_{x-y}\left( k \right)}{1+k}$$

19. Inverse Difference Normalized (IDN)

$$IDN=\sum_{k=0}^{N_{g}-1} \frac{p_{x-y}\left( k \right)}{1+(\frac{k}{N_{g}})}$$

20. Inverse Variance

$$inverse variance=\sum_{k=1}^{N_{g}-1} \frac{p_{x-y}\left( k \right)}{k^{2}}$$

21. Maximum Probability

$$maximum probability=max(p(i,j))$$

22. Sum Average

$$sum average=\sum_{k=2}^{{2N}_{g}} p_{x+y}\left( k \right)k$$

23. Sum Entropy

$$sum entropy=\sum_{k=2}^{{2N}_{g}} p_{x+y}\left( k \right){log}_{2}\left( p_{x+y}\left( k \right)+\epsilon\right)$$

24. Sum of Squares

$$sum squares=\sum_{i=1}^{N_{g}} \sum_{j=1}^{N_{g}} \left( i-\mu_{x} \right)^{2}p(i,j)$$

Gray Level Dependence Matrix (GLDM) Features

A Gray Level Dependence Matrix (GLDM) quantifies gray level dependencies in an image. A gray level dependency is defined as the number of connected voxels within distance $\delta$ that are dependent on the center voxel. A neighboring voxel with gray level $j$ is considered dependent on center voxel with gray level $i$ if $|i-j|\leq\alpha$. In a gray level dependence matrix $P(i,j)$ the ${(i,j)}^{th}$ element describes the number of times a voxel with gray level $i$ with $j$ dependent voxels in its neighborhood appears in image.

Let:

$N_{g}$ be the number of discreet intensity values in the image.

$N_{d}$ be the number of discreet dependency sizes in the image.

$N_{z}$ be the number of dependency zones in the image, which is equal to$\sum_{i=1}^{N_{g}} \sum_{j=1}^{N_{d}} \boldsymbol{P}(i,j)$.

$\boldsymbol{P}(i,j)$ be the dependence matrix.

$p(i,j)$ be the normalized dependence matrix, defined as $p\left( i,j \right)=\frac{\boldsymbol{P}(i,j)}{N_{z}}$.

1. Small Dependence Emphasis (SDE)

$$SDE=\frac{\sum_{i=1}^{N_{g}} \sum_{j=1}^{N_{d}} \frac{\boldsymbol{P}(i,j)}{j^{2}}}{N_{z}}$$

2. Large Dependence Emphasis (LDE)

$$LDE=\frac{\sum_{i=1}^{N_{g}} \sum_{j=1}^{N_{d}} \boldsymbol{P}(i,j)j^{2}}{N_{z}}$$

3. Gray Level Non-Uniformity (GLN)

$$GLN=\frac{\sum_{i=1}^{N_{g}} {(\sum_{j=1}^{N_{d}} \boldsymbol{P}(i,j))}^{2}}{N_{z}}$$

4. Dependence Non-Uniformity (DN)

$$DN=\frac{\sum_{j=1}^{N_{d}} {(\sum_{i=1}^{N_{g}} \boldsymbol{P}(i,j))}^{2}}{N_{z}}$$

5. Dependence Non-Uniformity Normalized (DNN)

$$DNN=\frac{\sum_{i=1}^{N_{d}} {(\sum_{j=1}^{N_{g}} \boldsymbol{P}(i,j))}^{2}}{{N_{z}}^{2}}$$

6. Gray Level Variance (GLV)

$$GLV=\sum_{i=1}^{N_{g}} \sum_{j=1}^{N_{d}} p\left( i,j \right)\left( i-\mu\right)^{2}, where \mu=\sum_{i=1}^{N_{g}} \sum_{j=1}^{N_{d}} ip\left( i,j \right)$$

7. Dependence Variance (DV)

$$DV=\sum_{i=1}^{N_{g}} \sum_{j=1}^{N_{d}} p\left( i,j \right)\left( j-\mu\right)^{2}, where \mu=\sum_{i=1}^{N_{g}} \sum_{j=1}^{N_{d}} jp\left( i,j \right)$$

8. Dependence Entropy (DE)

$$DE=-\sum_{i=1}^{N_{g}} \sum_{j=1}^{N_{d}} p(i,j){log}_{2}(p(i,j)+\epsilon)$$

9. Low Gray Level Emphasis (LGLE)

$$LGLE=\frac{\sum_{i=1}^{N_{g}} \sum_{j=1}^{N_{d}} \frac{\boldsymbol{P}(i,j)}{i^{2}}}{N_{z}}$$

10. High Gray Level Emphasis (HGLE)

$$HGLE=\frac{\sum_{i=1}^{N_{g}} \sum_{j=1}^{N_{d}} \boldsymbol{P}(i,j)i^{2}}{N_{z}}$$

11. Small Dependence Low Gray Level Emphasis (SDLGLE)

$$SDLGLE=\frac{\sum_{i=1}^{N_{g}} \sum_{j=1}^{N_{d}} \frac{\boldsymbol{P}(i,j)}{i^{2}j^{2}}}{N_{z}}$$

12. Small Dependence High Gray Level Emphasis (SDHGLE)

$$SDHGLE=\frac{\sum_{i=1}^{N_{g}} \sum_{j=1}^{N_{d}} \frac{\boldsymbol{P}(i,j)i^{2}}{j^{2}}}{N_{z}}$$

13. Large Dependence Low Gray Level Emphasis (LDLGLE)

$$LDLGLE=\frac{\sum_{i=1}^{N_{g}} \sum_{j=1}^{N_{d}} \frac{\boldsymbol{P}(i,j)j^{2}}{i^{2}}}{N_{z}}$$

14. Large Dependence High Gray Level Emphasis (LDHGLE)

$$LDHGLE=\frac{\sum_{i=1}^{N_{g}} \sum_{j=1}^{N_{d}} \boldsymbol{P}(i,j)i^{2}j^{2}}{N_{z}}$$

Gray Level Run Length Matrix (GLRLM) Features

A Gray Level Run Length Matrix (GLRLM) quantifies gray level runs, which are defined as the length in number of pixels, of consecutive pixels that have the same gray level value. In a gray level run length matrix $P(i,j|\theta)$, the ${(i,j)}^{th}$ element describes the number of runs with gray level $i$ and length $j$ occur in the image (ROI) along angle $\theta$.

Let:

- $N_{g}$ be the number of discreet intensity values in the image.
- $N_{r}$ be the number of discreet run lengths in the image.
- $N_{p}$ be the number of voxels in the image.
- $N_{r}(\theta)$ be the number of runs in the image along angle $\theta$, which is equal to $\sum_{i=1}^{N_{g}} \sum_{j=1}^{N_{r}} \boldsymbol{P}(i,j|\theta)$ and 1$\leq N_{r}(\theta)\leq N_{p}$.
- $\boldsymbol{P}(i,j|\theta)$ be the run length matrix for an arbitrary direction $\theta$.
- $p(i,j|\theta)$ be the normalized run length matrix, defined as $p\left( i,j | \theta\right)=\frac{\boldsymbol{P}(i,j|\theta)}{N_{r}(\theta)}$

1. Short Run Emphasis (SRE)

$$SRE=\frac{\sum_{i=1}^{N_{g}} \sum_{j=1}^{N_{r}} \frac{\boldsymbol{P}(i,j|\theta)}{j^{2}}}{N_{r}(\theta)}$$

2. Long Run Emphasis (SRE)

$$LRE=\frac{\sum_{i=1}^{N_{g}} \sum_{j=1}^{N_{r}} \boldsymbol{P}(i,j|\theta)j^{2}}{N_{r}(\theta)}$$

3. Gray Level Non-Uniformity (GLN)

$$GLN=\frac{\sum_{i=1}^{N_{g}} {(\sum_{j=1}^{N_{r}} \boldsymbol{P}(i,j|\theta))}^{2}}{N_{r}(\theta)}$$

4. Gray Level Non-Uniformity Normalized (GLNN)

$$GLNN=\frac{\sum_{i=1}^{N_{g}} {(\sum_{j=1}^{N_{r}} \boldsymbol{P}(i,j|\theta))}^{2}}{{N_{r}(\theta)}^{2}}$$

5. Run Length Non-Uniformity (RLN)

$$RLN=\frac{\sum_{j=1}^{N_{r}} {(\sum_{i=1}^{N_{g}} \boldsymbol{P}(i,j|\theta))}^{2}}{N_{r}(\theta)}$$

6. Run Length Non-Uniformity Normalized (RLNN)

$$RLNN=\frac{\sum_{j=1}^{N_{r}} {(\sum_{i=1}^{N_{g}} \boldsymbol{P}(i,j|\theta))}^{2}}{{N_{r}(\theta)}^{2}}$$

7. Run Percentage (RP)

$$RP=\frac{N_{r}(\theta)}{N_{p}}$$

8. Gray Level Variance (GLV)

$$GLV=\sum_{i=1}^{N_{g}} \sum_{j=1}^{N_{r}} p\left( i,j|\theta\right)\left( i-\mu\right)^{2}, where \mu=\sum_{i=1}^{N_{g}} \sum_{j=1}^{N_{r}} p\left( i,j|\theta\right)i$$

9. Run Variance (RV)

$$RV=\sum_{i=1}^{N_{g}} \sum_{j=1}^{N_{r}} p\left( i,j|\theta\right)\left( j-\mu\right)^{2}, where \mu=\sum_{i=1}^{N_{g}} \sum_{j=1}^{N_{r}} p\left( i,j|\theta\right)j$$

10. Run Entropy (RE)

$$RE=-\sum_{i=1}^{N_{g}} \sum_{j=1}^{N_{r}} p(i,j|\theta){log}_{2}(p(i,j|\theta)+\epsilon)$$

11. Low Gray Level Run Emphasis (LGLRE)

$$LGLRE=\frac{\sum_{i=1}^{N_{g}} \sum_{j=1}^{N_{r}} \frac{\boldsymbol{P}(i,j|\theta)}{i^{2}}}{N_{r}(\theta)}$$

12. High Gray Level Run Emphasis (HGLRE)

$$HGLRE=\frac{\sum_{i=1}^{N_{g}} \sum_{j=1}^{N_{r}} \boldsymbol{P}(i,j|\theta)i^{2}}{N_{r}(\theta)}$$

13. Short Run Low Gray Level Emphasis (SRLGLE)

$$SRLGLE=\frac{\sum_{i=1}^{N_{g}} \sum_{j=1}^{N_{r}} \frac{\boldsymbol{P}(i,j|\theta)}{i^{2}j^{2}}}{N_{r}(\theta)}$$

14. Short Run High Gray Level Emphasis (SRHGLE)

$$SRHGLE=\frac{\sum_{i=1}^{N_{g}} \sum_{j=1}^{N_{r}} \frac{\boldsymbol{P}(i,j|\theta)i^{2}}{j^{2}}}{N_{r}(\theta)}$$

15. Long Run Low Gray Level Emphasis (LRLGLE)

$$LRLGLE=\frac{\sum_{i=1}^{N_{g}} \sum_{j=1}^{N_{r}} \frac{\boldsymbol{P}(i,j|\theta)j^{2}}{i^{2}}}{N_{r}(\theta)}$$

16. Long Run High Gray Level Emphasis (LRHGLE)

$$LRHGLE=\frac{\sum_{i=1}^{N_{g}} \sum_{j=1}^{N_{r}} \boldsymbol{P}(i,j|\theta)i^{2}j^{2}}{N_{r}(\theta)}$$

Gray Level Size Zone Matrix (GLSZM) Features

A Gray Level Size Zone (GLSZM) quantifies gray level zones in an image. A gray level zone is defined as a number of connected voxels that share the same gray level intensity. A voxel is considered connected if the distance is 1 according to the infinity norm (26-connected region in a 3D, 8-connected region in 2D). In a gray level size zone matrix $\boldsymbol{P}(i,j)$ the $(i,j)$th element equals the number of zones with gray level $i$ and size $j$ appear in image. Contrary to GLCM and GLRLM, the GLSZM is rotation independent, with only one matrix calculated for all directions in the ROI.

Let:

- $N_{g}$ be the number of discreet intensity values in the image.
- $N_{s}$ be the number of discreet zone sizes in the image.
- $N_{p}$ be the number of voxels in the image.
- $N_{z}$ be the number of zones in the ROI, which is equal to $\sum_{i=1}^{N_{g}} \sum_{j=1}^{N_{s}} \boldsymbol{P}(i,j)$ and $1\leq N_{z}\leq N_{p}$.
- $\boldsymbol{P}(i,j)$ be the size zone matrix.
- $p(i,j)$ be the normalized size zone matrix, defined as $p\left( i,j \right)=\frac{\boldsymbol{P}(i,j)}{N_{z}}$.

1. Small Area Emphasis (SAE)

$$SAE=\frac{\sum_{i=1}^{N_{g}} \sum_{j=1}^{N_{s}} \frac{\boldsymbol{P}(i,j)}{j^{2}}}{N_{z}}$$

2. Large Area Emphasis (LAE)

$$LAE=\frac{\sum_{i=1}^{N_{g}} \sum_{j=1}^{N_{s}} \boldsymbol{P}(i,j)j^{2}}{N_{z}}$$

3. Gray Level Non-Uniformity (GLN)

$$GLN=\frac{\sum_{i=1}^{N_{g}} {(\sum_{j=1}^{N_{s}} \boldsymbol{P}(i,j))}^{2}}{N_{z}}$$

4. Gray Level Non-Uniformity Normalized (GLNN)

$$GLNN=\frac{\sum_{i=1}^{N_{g}} {(\sum_{j=1}^{N_{s}} \boldsymbol{P}(i,j))}^{2}}{{N_{z}}^{2}}$$

5. Size-Zone Non-Uniformity (SZN)

$$SZN=\frac{\sum_{j=1}^{N_{s}} {(\sum_{i=1}^{N_{g}} \boldsymbol{P}(i,j))}^{2}}{N_{z}}$$

6. Size-Zone Non-Uniformity Normalized (SZNN)

$$SZNN=\frac{\sum_{j=1}^{N_{s}} {(\sum_{i=1}^{N_{g}} \boldsymbol{P}(i,j))}^{2}}{{N_{z}}^{2}}$$

7. Zone Percentage (ZP)

$$ZP=\frac{N_{z}}{N_{p}}$$

8. Gray Level Variance (GLV)

$$GLV=\sum_{i=1}^{N_{g}} \sum_{j=1}^{N_{s}} p(i,j){(i-\mu)}^{2}$$

$$\mu=\sum_{i=1}^{N_{g}} \sum_{j=1}^{N_{s}} p(i,j)i$$

9. Zone Variance (ZV)

$$ZV=\sum_{i=1}^{N_{g}} \sum_{j=1}^{N_{s}} p(i,j){(j-\mu)}^{2}$$

$$\mu=\sum_{i=1}^{N_{g}} \sum_{j=1}^{N_{s}} p(i,j)j$$

10. Zone Entropy (ZE)

$$ZE=-\sum_{i=1}^{N_{g}} \sum_{j=1}^{N_{s}} p(i,j){log}_{2}(p(i,j)+\epsilon)$$

11. Low Gray Level Zone Emphasis (LGLZE)

$$LGLZE=\frac{\sum_{i=1}^{N_{g}} \sum_{j=1}^{N_{s}} \frac{\boldsymbol{P}(i,j)}{i^{2}}}{N_{z}}$$

12. High Gray Level Zone Emphasis (HGLZE)

$$HGLZE=\frac{\sum_{i=1}^{N_{g}} \sum_{j=1}^{N_{s}} \boldsymbol{P}(i,j)i^{2}}{N_{z}}$$

13. Small Area Low Gray Level Emphasis (SALGLE)

$$SALGLE=\frac{\sum_{i=1}^{N_{g}} \sum_{j=1}^{N_{s}} \frac{\boldsymbol{P}(i,j)}{i^{2}j^{2}}}{N_{z}}$$

14. Small Area High Gray Level Emphasis (SAHGLE)

$$SAHGLE=\frac{\sum_{i=1}^{N_{g}} \sum_{j=1}^{N_{s}} \frac{\boldsymbol{P}(i,j)i^{2}}{j^{2}}}{N_{z}}$$

15. Large Area Low Gray Level Emphasis (LALGLE)

$$LALGLE=\frac{\sum_{i=1}^{N_{g}} \sum_{j=1}^{N_{s}} \frac{\boldsymbol{P}(i,j)j^{2}}{i^{2}}}{N_{z}}$$

16. Large Area High Gray Level Emphasis (LAHGLE)

$$LAHGLE=\frac{\sum_{i=1}^{N_{g}} \sum_{j=1}^{N_{s}} \boldsymbol{P}(i,j){i^{2}j}^{2}}{N_{z}}$$

Neighboring Gray Tone Difference Matrix (NGTDM) Features

A Neighboring Gray Tone Difference Matrix quantifies the difference between a gray value and the average gray value of its neighbors within distance $\delta$. The sum of absolute differences for gray level $i$ is stored in the matrix. Let $\boldsymbol{X}_{gl}$ be a set of segmented voxels and $x_{gl}(j_{x},j_{y},j_{z})\in\boldsymbol{X}_{gl}$ be the gray level of a voxel at position $(j_{x},j_{y},j_{z})$, then the average gray level of the neigbourhood is:

$$\bar{A}_{i}=\bar{A}\left( j_{x},j_{y},j_{z} \right)=\frac{1}{W}\sum_{k_{x}=-\delta}^{\delta} \sum_{k_{y}=-\delta}^{\delta} \sum_{k_{z}=-\delta}^{\delta} x_{gl}(j_{x}+k_{x},j_{y}+k_{y},j_{z}+k_{z}), where\left( k_{x},k_{y},k_{z} \right)\neq\left( 0,0,0 \right) and x_{gl}\left( j_{x}+k_{x},j_{y}+k_{y},j_{z}+k_{z} \right)\in\boldsymbol{X}_{gl}.$$

Here, $W$ is the number of voxels in the neighborhood that are also in $\boldsymbol{X}_{gl}$.

Let:

- $n_{i}$ be the number of voxels in $X_{gl}$ with gray level $i$.
- $N_{v,p}$ be the total number of voxels in $X_{gl}$ and equal to $\sum n_{i}$ (i.e., the number of voxels with a valid region; at least 1 neighbor). $N_{v,p}\leq N_{p}$, where $N_{p}$ is the total number of voxels in the ROI.
- $p_{i}$ be the gray level probability and equal to $n_{i}/N_{v}$.
- $s_{i}=\left\{ \begin{aligned} \sum_{n_{i}} \left| i-\bar{A}_{i} \right| \mathrm{for} n_{i}\neq0 \\ 0 \mathrm{for} n_{i}=0 \end{aligned} \right.$be the sum of absolute differences for gray level $i$.
- $N_{g}$ be the number of discreet gray levels.
- $N_{g,p}$ be the number of gray levels where $p_{i}\neq0$.

1. Coarseness

$$coarseness=\frac{1}{\sum_{i=1}^{N_{g}} p_{i}s_{i}}$$

2. Contrast

$$contrast=\left( \frac{1}{N_{g,p}\left( N_{g,p}-1 \right)}\sum_{i=1}^{N_{g}} \sum_{j=1}^{N_{g}} p_{i}p_{j}\left( i-j \right)^{2} \right)\left( \frac{1}{N_{v,p}}\sum_{i=1}^{N_{g}} s_{i} \right), where p_{i}\neq0, p_{j}\neq0$$

3. Busyness

$$busyness=\frac{\sum_{i=1}^{N_{g}} p_{i}s_{i}}{\sum_{i=1}^{N_{g}} \sum_{j=1}^{N_{g}} |ip_{i}-jp_{j}|}, where p_{i}\neq0, p_{j}\neq0$$

4. Complexity

$$complexity=\frac{1}{N_{v,p}}\sum_{i=1}^{N_{g}} \sum_{j=1}^{N_{g}} |i-j|\frac{p_{i}s_{i}+p_{j}s_{j}}{p_{i}+p_{j}}, wherep_{i}\neq0, p_{j}\neq0$$

5. Strength

$$strength=\frac{\sum_{i=1}^{N_{g}} \sum_{j=1}^{N_{g}} (p_{i}+p_{j}){(i-j)}^{2}}{\sum_{i=1}^{N_{g}} s_{i}}, where p_{i}\neq0, p_{j}\neq0$$

**Time domain features**

Time domain features were extracted based on each spatial domain feature except for the common shape features. Here ${dyn}_{0}$, ${dyn}_{1}$, ${dyn}_{2}$ and ${dyn}_{3}$ represent the mask phase, first enhanced phase, second enhanced phase and third enhanced phase, respectively.

Time domain features indicate the mean, variance, skewness, kurtosis and entropy of the time-varying curves (totally 3348 features).

1. Mean

$$mean({DYN=[dyn}_{1}\ldots{dyn}_{N}])=\frac{1}{N}\sum_{i=1}^{N} {dyn}_{i}$$

2. Variance

$$variance({DYN=[dyn}_{1}\ldots{dyn}_{N}])=\frac{1}{N-1}\sum_{i=1}^{N} {({dyn}_{i}-\bar{DYN})}^{2}$$

3. Skewness

$$skewness\left( {DYN=[dyn}_{1}\ldots{dyn}_{N}] \right)=\frac{1}{N}\sum_{i=1}^{N} \left[ \frac{{dyn}_{i}-\bar{DYN}}{\sigma} \right]^{3}$$

4. Kurtosis

$$kurtosis\left( {DYN=[dyn}_{1}\ldots{dyn}_{N}] \right)=\left\{ \frac{1}{N}\sum_{i=1}^{N} \left[ \frac{{dyn}_{i}-\bar{DYN}}{\sigma} \right]^{4} \right\}-3$$

here $N=4$ corresponds to 4 DCE phases, $\sigma=\sqrt{Var({dyn}_{1}\ldots{dyn}_{N})}$ is the distribution’s standard deviation.
